# Mangelernährung bei Patienten mit Lungenkrebs: Einfluss des präoperativen Ernährungsstatus auf das postoperative Outcome bei kurativ operierten Patienten mit nichtkleinzelligem Lungenkarzinom (NSCLC)

**DOI:** 10.1007/s00104-026-02466-5

**Published:** 2026-02-25

**Authors:** T. Rabenhold, M. Krüger, W. Schütte, M. Möller, N. M. Dörr-Jerat

**Affiliations:** 1 Klinik f. Thoraxchirurgie, Krankenhaus Martha-Maria Halle-Dölau, Röntgenstr. 1, 06120 Halle (Saale), Deutschland; 2 Medizinische Fakultät, Martin-Luther-Universität Halle-Wittenberg, Halle-Wittenberg, Deutschland

**Keywords:** NSCLC, Ernährungsstatus, Mangelernährung, Postoperatives Outcome, Überleben, NSCLC, Nutritional status, Malnutrition, Postoperative outcome, Survival

## Abstract

**Hintergrund:**

Mangelernährung stellt für Patienten mit nichtkleinzelligem Lungenkarzinom (NSCLC) ein Risiko dar. Mit reduziertem Ernährungsstatus gehen funktionelle Verschlechterungen und perioperative Komplikationen einher. Infolge werden Therapieerfolg und Prognose beeinträchtigt.

**Methodik:**

Es wurde eine retrospektive, monozentrische Kohortenstudie unter Einschluss von 197 NSCLC-Patienten, die 2015 bis 2024 in kurativer Intention operiert wurden, durchgeführt. Ziel war es, den Zusammenhang zwischen präoperativem Ernährungsstatus, postoperativem Outcome und Langzeitverlauf bei NSCLC-Patienten herauszustellen.

**Ergebnisse:**

Gemäß altersadaptiertem BMI waren präoperativ 28,4 % der Patienten untergewichtig, 35,5 % normalgewichtig und 36,1 % übergewichtig. Etwa ein Drittel des Kollektivs hatte im NRS ≥ 3 Punkte; 65 % hatte im GMS ≥ 3 Punkte (jeweils Risiko für Malnutrition); 44,2 % imponierten mit einem CAR-Wert ≥ 0,144. Schwere postoperative Komplikationen (CDK ≥ °III) waren mit NRS ≥ 3, GMS ≥ 3 und CAR ≥ 0,144 assoziiert. Für Patienten mit auffälligem NRS und GMS ergab sich ein höheres Rezidiv- und Sterberisiko. Eine Erhöhung der CAR um den Wert 1 war mit einem 41 % höheren Sterberisiko verbunden.

**Diskussion:**

Der präoperative Ernährungsstatus beeinflusste das Outcome nach kurativer OP. Aufgrund besonderer prädiktiver Relevanz einzelner Parameter sollten Ernährungsscreenings multiparametrisch erfolgen. Weitere Studien sind notwendig, um die Ergebnisse im Sinne prähabilitativer Ernährungskonzepte im klinischen Alltag nutzbar zu machen.

**Graphic abstract:**

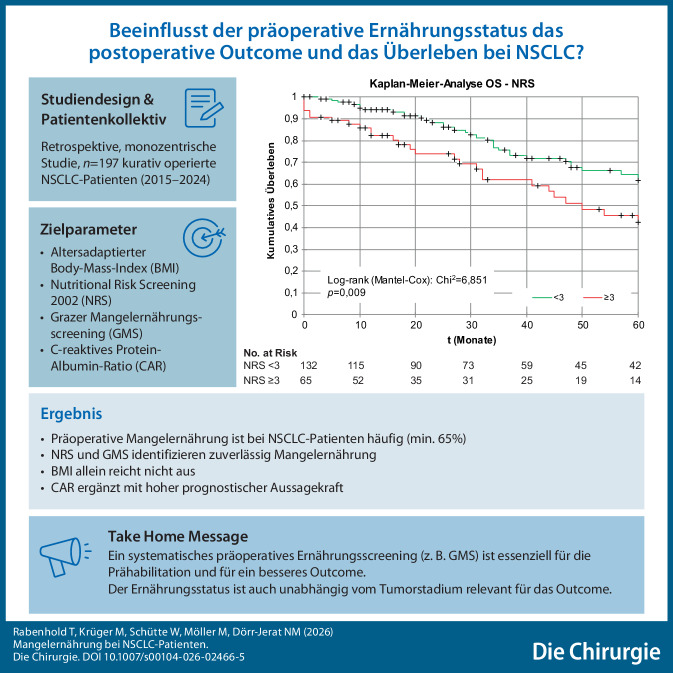

## Abstract (en)

**Background:**

Malnutrition poses a significant risk for patients with non-small cell lung cancer (NSCLC). A reduced nutritional status is associated with functional decline and perioperative complications, thereby impairing treatment success and prognosis.

**Methods:**

A retrospective single-center cohort study including 197 NSCLC patients who underwent curative surgery between 2015 and 2024 was conducted. The aim was to examine the relationship between preoperative nutritional status, postoperative outcome and long-term prognosis in NSCLC patients.

**Results:**

Based on age-adjusted body mass index (BMI) 28.4% of patients were underweight, 35.5% normal weight and 36.1% overweight. Approximately one third of the patients had a nutritional risk scoring (NRS) ≥ 3 and 65% a Grazer malnutrition screening (GMS) ≥ 3, each indicating a risk of malnutrition. A C-reactive protein-albumin ratio (CAR) ≥ 0.144 was observed in 44.2% of patients. Severe postoperative complications (Clavien-Dindo classification, CDC ≥ °III) were associated with NRS ≥ 3, GMS ≥ 3 and CAR ≥ 0.144. Patients with elevated NRS and GMS scores showed a higher risk of recurrence and mortality. An increase in CAR by 1 was associated with a 41% higher mortality risk.

**Conclusion:**

The preoperative nutritional status influenced postoperative outcomes after curative surgery. Given the predictive relevance of individual parameters, nutritional screening should be performed multiparametrically. Further studies are needed to translate these findings into clinical practice and prehabilitation-based nutritional strategies.

Mangelernährung stellt hinsichtlich der operativen Therapie des nichtkleinzelligen Lungenkarzinoms (NSCLC) einen relevanten Risikofaktor dar. Sie bedingt nicht nur erhöhte Morbidität und Mortalität, sondern beeinträchtigt auch die körperliche Leistungsfähigkeit, die Lebensqualität und die Therapieverträglichkeit [14]. Etwa 70 % aller NSCLC-Patienten sind mindestens 1‑mal im Laufe ihrer Erkrankung von Mangelernährung betroffen [11].

Um Ursachen und Folgen von Mangelernährung entgegenzuwirken, haben sich multimodale Maßnahmen zur präoperativen Patientenvorbereitung entwickelt. Dieses Konzept der ernährungsbezogenen Prähabilitation soll den Ernährungsstatus vor Therapiebeginn stabilisieren oder verbessern [6]. Hinreichende Belege, dass gezielte präoperative Ernährungstherapie perioperative Komplikationen und die Krankenhausverweildauer senkt, wurden auch in Studien mit anderen Tumorentitäten erbracht [7].

Die vorliegende Studie beschäftigt sich mit dem Einfluss des präoperativen Ernährungsstatus auf das postoperative Outcome bei NSCLC-Patienten mit dem Ziel der Verbesserung von präoperativen Ernährungsscreenings im klinischen Alltag.

## Methodik

### Stichprobe

In einer retrospektiven, monozentrischen Kohortenstudie wurden 197 NSCLC-Patienten untersucht, die zwischen 2015 und 2024 kurativ operiert wurden. Ziel war es, den Zusammenhang von Mangelernährung und postoperativem Outcome nach NSCLC-Resektion zu analysieren. Einschlusskriterien waren Volljährigkeit, gesicherte NSCLC-Diagnose und kurative Operation; ausgeschlossen wurden palliative intendierte OPs und fehlende oder unvollständige Nachsorge.

### Datenerhebung

Der Ernährungsstatus wurde über Body-Mass-Index (BMI), Nutritional Risk Screening 2002 (NRS), Grazer Mangelernährungsscreening (GMS) und C‑reaktives Protein-Albumin-Ratio (CAR) erfasst [12, 18, 22]. Relevante Komorbiditäten wurden nach Charlson-Comorbidity-Index (CCI) bepunktet [3]. Postoperative Komplikationen wurden mittels Clavien-Dindo-Klassifikation (CDK) dokumentiert [5]. Das Patientenkollektiv wurde weiterhin hinsichtlich der Krankenhausverweildauer („length of stay“ [LOS]) untersucht.

### Statistische Datenanalyse

Zur Analyse der Abhängigkeiten zweier Variablen wurde der Chi^2^-Test eingesetzt. Normalverteilte Daten wurden mit t‑Test, nicht-normalverteilte mit Mann-Whitney-U-Test geprüft. Korrelations- und Regressionsanalysen wurden entsprechend durchgeführt. Überlebenszeiten (rezidivfreies Überleben [„recurrence-free survival“, RFS], 2‑ und 5‑Jahres- Überlebensraten [„overall survival“, OS]) wurden mittels Kaplan-Meier- und Log-Rank-Test analysiert. RFS wurde definiert als Zeit vom Tag der OP bis zum Datum eines Rezidivs; OS als die Zeit vom Tag der OP bis zum Datum des Todes unabhängig von der Todesursache. Eine ROC-Analyse wurde durchgeführt, um den Cut-off der CAR zur Vorhersage von RFS und OS zu bestimmen. Signifikanz wurde über 95 %-Konfidenzintervalle und *p* = 0,05 (5 %) beurteilt.

## Ergebnisse

### Patientenkollektiv

Die Tab. 1 zeigt die Charakteristika des Patientenkollektivs. Es wurden 197 NSCLC-Patienten wurden einbezogen; 130 (66,0 %) Patienten waren männlich und 67 (34,0 %) weiblich. Das Durchschnittsalter zum OP-Zeitpunkt betrug 66,4 Jahre (SD = 9,8), wobei das mediane Alter bei 67 Jahren (Range: 21–85) lag.

**Tab. 1:** Charakterisierung der Gesamtstichprobe (*n* = 197)

*Alter (Jahre)*	
Median (Range)	67 (21–85)
*Geschlechterverteilung (%)*	
Männlich (*n*)	66,0 (130)
Weiblich (*n*)	34,0 (67)
*Raucherstatus, SMKSTAT2 (%)*	
1 tägliche Raucher	42,6
2 gelegentliche Raucher	1,0
3 Ex-Raucher	42,2
4 Nie‑/Nichtraucher	13,2
5/9 unbekannt/unklar, ob geraucht	1,0
*Lungenfunktion (Durchschnitte in %)*	
FEV1 (SD)	79,9 (19,1)
TLCO (SD)	77,1 (19,4)
*Komorbiditäten, CCI (%)*	
0–4 Punkte	25,4
5–7 Punkte	51,8
8–10 Punkte	19,3
> 10 Punkte	3,5
Punktedurchschnitt (SD)	6,1 (2,2)
*Tumorgröße (mm), Durchschnitt (SD)*	43,8 (22)
*Histopathologie (%)*	
Adenokarzinome	47,7
Plattenepithelkarzinome	36,0
Neuroendokrine Tumoren/Karzinome	10,2
Sonstige	6,1
*Tumorstadien (%)*	
I	31,0
II	34,0
III	33,0
IV	2,0
*Immunhistochemie (%)*	
ALK	1,0
EGFR	3,0
PD-L1	21,8
Mehrere	4,1
Keine	70,1

*n* Anzahl, *SD* „standard deviation“, Standardabweichung, *SMKSTAT2* „Smoking Status Recode 2“ [9], *FEV1* funktionelle Einsekundenkapazität, *TLCO* Diffusionskapazität, *CCI* Charlson-Comorbidity Index, *ALK* anaplastische Lymphomkinase, *EGFR* „epidermal growth factor receptor“, *PD-L1* „programmed death-ligand 1“

Adenokarzinome (47,7 %) waren am häufigsten, gefolgt von Plattenepithelkarzinomen (36 %) und neuroendokrinen Tumoren (10,2 %). Die Tumorgröße betrug durchschnittlich 43,8 mm (SD = 22; Range: 3–120). In 69 % der Fälle lag ein frühes lokal fortgeschrittenes Tumorstadium (≥ II) vor. Die meisten Patienten befanden sich im Tumorstadium IIIA (25,4 %). Neben den Stadien I–IIIA wurden auch atypisch kurativ therapierbare Tumorstadien (IIIB–IV) berücksichtigt; 7,1 % erhielten eine neoadjuvante Therapie.

Es waren 21,8 % PD-L1-positive NSCLC. Etwa 3 % der Tumoren wiesen eine EGFR-Mutation auf; 1 % eine ALK-Translokation.

### Präoperativer Ernährungsstatus und postoperatives Outcome

Gemäß altersadaptierten BMI-Normwerten waren 28,4 % der Patienten untergewichtig, 35,5 % normalgewichtig und 36,1 % übergewichtig. Die meisten Untergewichtigen gehörten zur Altersgruppe 51 bis 60 Jahre; die meisten Übergewichtigen waren im Alter von 61 bis 70 Jahren; 33 % hatte im NRS einen Punktwert ≥ 3 Punkte. Das GMS identifizierte 65 % mit ≥ 3 Punkten (jeweils Risko für Malnutrition). Ein niedrigerer BMI war dabei mit höherem Punktwert im NRS assoziiert (ρ = −0,35 [−0,47–−0,21]; *p* < 0,0001).

**Abb. 1:**
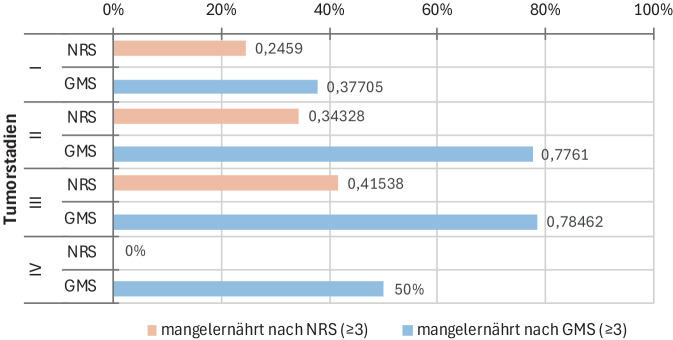
Mangelernährungsprävalenz nach Tumorstadium. *NRS* Nutritional Risk Screening, *GMS* Grazer Mangelernährungsscreening

Die Mangelernährungsprävalenz steigt in den Tumorstadien I–III mit höherem Stadium (Abb. 1). GMS (τ = 0,29 [0,21–0,38]; *p* < 0,0001) und CAR (τ = 0,18 [0,08–0,26]; *p* = 0,002) korrelierten mit dem Tumorstadium; NRS (τ = 0,11 [0,01–0,2]; *p* = 0,115) nicht.

Die durchschnittliche CAR belief sich auf 0,54 (SD = 1,12; Range: 0,06–9,45); 87 Patienten (44,2 %) überschritten den mittels ROC-Verfahren errechneten Cut-off von ≥ 0,144 (SEN = 0,65; SPE = 0,62; AUC=0,6 [0,51–0,69]; *p* = 0,013; PPV = 0,45 [0,35–0,55]; NPV = 0,79 [0,7–0,86]).

Patienten mit neoadjuvanter Therapie hatten höhere Punktwerte bei NRS und GMS sowie einen niedrigeren BMI; allerdings auch niedrigere CAR-Werte (Tab. 2). Eine statistische Relevanz bestand nur zum BMI (U = 2259,5; Var[U] = 58.409,5; *p* = 0,04; z = 2,023; r = 0,226).

**Tab. 2:** Ernährungsstatus mit und ohne neoadjuvante Therapie

	Mit neoadjuvanter Therapie	Ohne neoadjuvante Therapie	Differenz (%)
NRS	2,550	2,073	*18,7*
GMS	3,350	2,904	*13,3*
BMI	24,371 kg/m^2^	26,969 kg/m^2^	*9,6*
CAR	0,423 mg/g	0,558 mg/g	*24,2*

*NRS* Nutritional Risk Screening 2002, *GMS* Grazer Mangelernährungsscreening, *BMI* Body-Mass-Index, *CAR* C-reaktives Protein-Albumin-Ratio

In 59,4 % der Fälle lagen Malnutrition-begünstigende Komorbiditäten vor. Am häufigsten waren COPD (27,4 %) und Diabetes mellitus (20,8 %), gefolgt von Lebererkrankungen und/oder chronischem Alkoholabusus (zusammen 13,7 %) sowie gastrointestinale Erkrankungen (12,2 %).

Bei 80,7 % des Patientenkollektivs kam es zu Abweichungen vom normalen postoperativen Verlauf (CDK ≥ °I). Schwere Komplikationen (CDK ≥ °III) mit hohem Interventionsbedarf erlitten 37,1 % (°IIIa: 20,8 %; °IIIb: 8,2 %; °IVa: 2,5 %; °IVb: 2,5 %; °V: 3,1 %).

Patienten mit NRS ≥ 3 Punkte hatten eine 4,16fach höhere Odds für schwere Komplikationen als Patienten mit < 3 Punkten (OR = 4,16 [2,23–7,76]; Chi^2^ = 20,8; *p* < 0,0001; V = 0,325). NRS (ρ = 0,34 [0,21–0,46]; *p* < 0,0001) und GMS (ρ = 0,31 [0,17–0,43]; *p* < 0,0001) zeigten deutliche positive Korrelationen mit der Schwere der postoperativen Komplikationen. Ferner wiesen Patienten mit schweren Komplikationen präoperativ höhere CAR-Werte auf als jene ohne schwere Komplikationen (U = 1356,5; Var[U] = 37.426,7; *p* = 0,018; z = −2,357; r = 0,213). Ein höheres präoperatives Serumalbumin zeigte sich protektiv (OR = 0,92 [0,86–0,99]; *p* = 0,028).

Andere untersuchte Variablen (z. B. BMI [U = 2121; Var(U) = 37.468,2; *p* = 0,11; z = 1,589; r = 0,143]) zeigten keinen Einfluss auf die Schwere postoperativer Komplikationen.

Einfluss auf die LOS hatten v. a. NRS (ρ = 0,290 [0,15–0,42]; *p* < 0,0001) und GMS (ρ = 0,34 [0,21–0,46]; *p* < 0,0001). Bei Patienten mit CAR ≥ 0,144; NRS ≥ 3 und GMS ≥ 3 wurden eine Intensivdauer von 5,6 Tagen und eine LOS von 15 bis 17 Tagen prognostiziert. Bei unauffälligem Ernährungsstatus reduzierten sich Intensivdauer (auf 1,8 bis 2,1 Tage) und LOS (auf 6 bis 7 Tage).

### Langzeitverlauf

#### Rezidivfreies Überleben

**Abb. 2:**
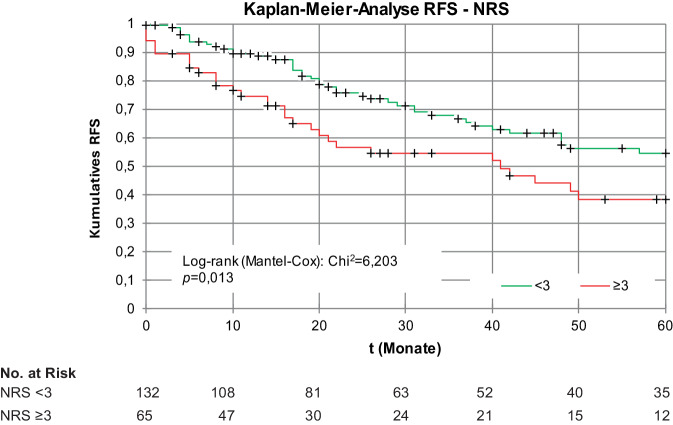
Kaplan-Meier-Analyse RFS in Abhängigkeit des NRS. *RFS* „recurrence-free survival“, *NRS* Nutritional Risk Screening, *t* Zeit

Die Abb. 2 stellt das RFS in Abhängigkeit der NRS-Gruppen (Grp) (< 3 vs. ≥ 3) dar. Während Grp < 3 nach 24 Monaten zu 75,6 % und nach 60 Monaten zu 54,4 % kein Rezidiv hatte, blieben in Grp ≥ 3 56,5 % (24 Monate) bzw. 38,4 % (60 Monate) rezidivfrei. Das mittlere RFS betrug 42,4 vs. 34,7 Monate. Für Grp ≥ 3 ist das Rezidivrisiko 38,7 % höher als für Grp < 3 (Cox: HR = 1,387 [0,53–3,61]; *p* = 0,45).

**Abb. 3:**
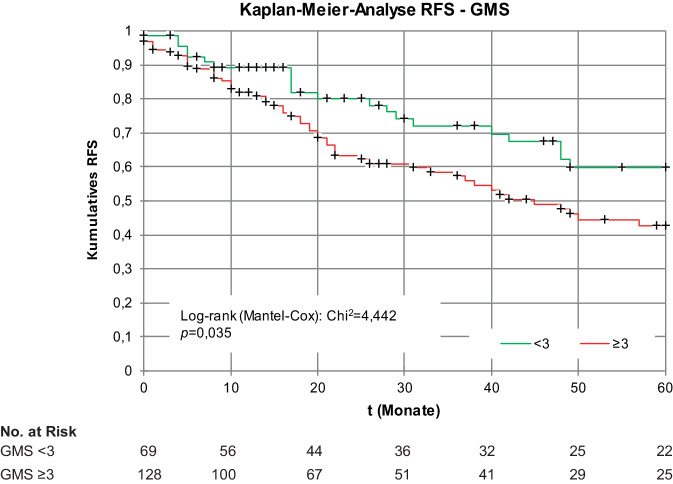
Kaplan-Meier-Analyse RFS in Abhängigkeit des GMS. *RFS* „recurrence-free survival“, *GMS* Grazer Mangelernährungsscreening, *t* Zeit

Auch für das GMS zeigten sich im Vergleich (< 3 vs. ≥ 3) Unterschiede (Abb. 3); 24 Monate nach OP sind in Grp ≥ 3 63,2 % ohne Rezidiv; in Grp < 3 80,1 %. Das RFS nach 60 Monaten von 59,9 % vs. 42,8 % hebt diesen Unterschied hervor. Das mittlere RFS betrug 37,1 vs. 39,3 Monate. Grp ≥ 3 präsentierte sich mit einem 22,6 % höheren Rezidivrisiko (Cox: HR = 1,226 [0,87–1,73]; *p* = 0,23).

Ferner ist ein Anstieg der CAR um 1 mit einem 29 % höheren Rezidivrisiko verbunden (Cox: HR = 1,29 [1,05–1,58]; *p* = 0,014).

#### Gesamtüberleben

**Abb. 4:**
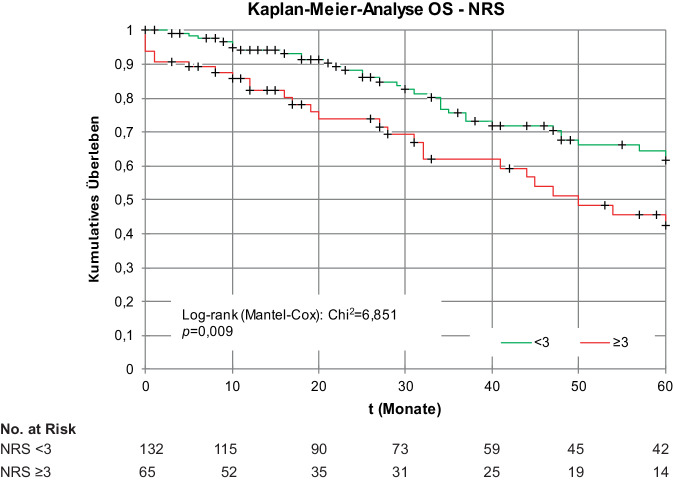
Kaplan-Meier-Analyse OS in Abhängigkeit des NRS. *OS* „overall survival“, *NRS* Nutritional Risk Screening; *t* Zeit

**Abb. 5:**
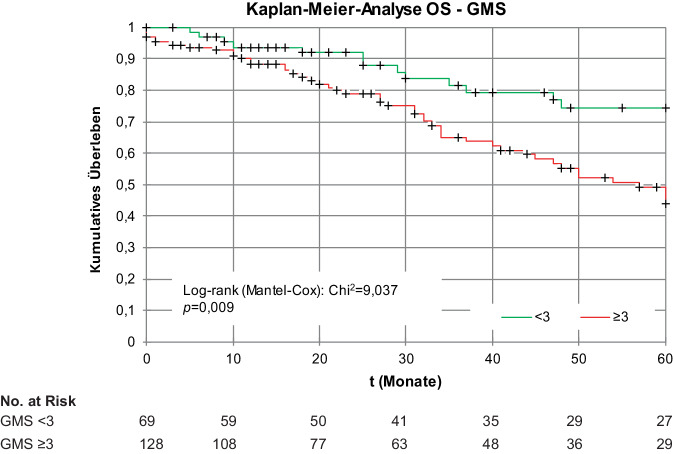
Kaplan-Meier-Analyse OS in Abhängigkeit des GMS. *OS* „overall survival“, *GMS* Grazer Mangelernährungsscreening, *t* Zeit

Die Abb. 4 zeigt das OS anhand der NRS-Gruppen (< 3 vs. ≥ 3) (90-Tage-Mortalität 0,8 % vs. 9,2 %; 2 JÜR: 88,2 % vs. 73,8 %; 5 JÜR: 61,6 % vs. 42,4 %). Ein NRS ≥ 3 bedingte ein 70 % höheres Sterberisiko (Cox: HR = 1,7 [1,03–2,82]; *p* = 0,04). Die Abb. 5 zeigt den Vergleich der GMS-Gruppen(< 3 vs. ≥ 3) (90-Tage-Mortalität 0 % vs. 5,4 %; 2 JÜR 91,9 % vs. 78,8 %; 5 JÜR 74,4 % vs. 43,9 %). Im Vergleich konnte für Grp ≥ 3 ein 25,3 % höheres Sterberisiko ermittelt werden (Cox: HR = 1,253 [0,54–2,92]; *p* = 0,6).

**Abb. 6:**
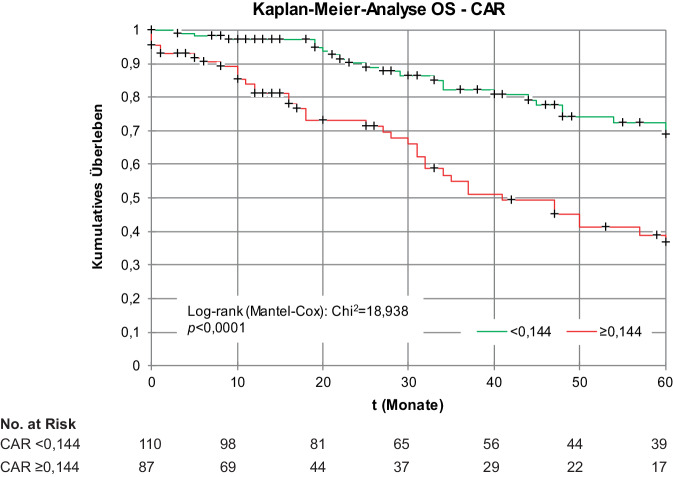
Kaplan-Meier-Analyse OS in Abhängigkeit der CAR. *OS* „overall survival“, *CAR* C-reaktives Protein-Albumin-Ratio, *t* Zeit

Der OS-Vergleich der CAR-Gruppen (< 0,144 vs. ≥ 0,144) zeigte sich prädiktiv (90-Tage-Mortalität 0,8 % vs. 4,6 %; 2 JÜR: 90,1 % vs. 73,2 %; 5 JÜR: 68,7 % vs. 36,7 %) (Abb. 6). Ein Anstieg der CAR um 1 ging mit einem 41 % höheren Sterberisiko einher (Cox: HR = 1,41 [1,14–1,74]; *p* = 0,001).

Weiterhin korrelierten folgende (nichternährungsbezogenen) Variablen mit dem OS:

- Je Lebensjahr steigt das Sterberisiko um 5,5 % (Cox: HR = 1,06 [1–1,11]; *p* = 0,049).
- Fortgeschrittene Tumorstadien (≥ III) erhöhen das Sterberisiko (HR = 5,15 [2,46–10,78]; *p* < 0,0001), wobei keine Interaktion mit dem Ernährungsstatus besteht (*p* = 0,848).
- Neoadjuvante Therapien hatten keinen Einfluss auf den Ernährungsstatus als Prädiktor (*p* = 0,46).
- Höhere präoperative TLCO-Werte wirken sich protektiv aus (Cox: HR = 0,97 [0–0,99]; *p* = 0,009).
- Weibliches Geschlecht ist mit geringerem Sterberisiko verbunden (Cox: HR = 0,46 [0–0,94]; *p* = 0,034).

## Diskussion

In unserer Studie wurde der Ernährungsstatus bei NSCLC-Patienten erstmals in Deutschland multimodal erfasst. Präoperative Mangelernährung zeigte sich dabei als hochprävalente Erkrankung: 28,4 % der Patienten waren nach altersadaptiertem BMI untergewichtig. Gemäß NRS lag die Prävalenzrate bei 33 %, nach GMS sogar bei 65 %. Zudem zeigten 44,2 % präoperativ eine erhöhte CAR. Diese Häufigkeiten decken sich mit früheren Untersuchungen, die eine Prävalenz von 30–70 % beschreiben [11, 17]. Die Prävalenz ist dabei abhängig vom verwendeten Screeningverfahren:

BMI-basierte Ansätze liefern eher konservative Ergebnisse, während GMS oder CAR mehr Patienten als „at risk“ identifizieren [14]. Dabei können auch Patienten mit Normal- oder Übergewicht betroffen sein. Dieser Zustand, welcher als „sarkopenische Adipositas“ bezeichnet wird, beschreibt ein Syndrom, bei dem vermehrte Fettmasse mit Verlust von Muskelmasse und -funktion einhergeht, wodurch ein Missverhältnis der Körperzusammensetzung entsteht. Mehrere Studien zeigten, dass der BMI allein keine Information über Körperverteilungsmerkmale liefert [2, 8]. Auch deshalb entschieden wir uns für umfassendere Ernährungsscreenings.

Internationale Unterschiede der Mangelernährungsprävalenz sind durch ethnische, sozioökonomische und methodische Faktoren erklärbar. Europäische Kohorten berichten meist Raten um 35–45 %, asiatische teils über 50 % [13, 27]. Ein universeller „Goldstandard“ für Ernährungsscreenings konnte bislang nicht etabliert werden, auch weil keine einheitliche Definition für Mangelernährung existiert [2]. Fehlende Screeningstandards erschweren die Vergleichbarkeit, weshalb die „American Society for Parenteral and Enteral Nutrition“ (ASPEN) den Einsatz validierter, populationsspezifischer Screenings nahelegt [21, 27].

Unsere Untersuchung nutzte BMI, NRS, GMS und CAR, wodurch entgegen vorheriger Studien mit ähnlichem Kollektiv die CAR komplementär zu Ernährungsscreenings als eigenständiger Marker verwendet wurde [16, 20]. Das NRS gilt nach ASPEN als am besten validiertes Screening bei onkologischen Patienten [21]. Die höchste Sensitivität wies das GMS auf (Prävalenz 65 %), ähnlich vorherigen Studien (SEN > 0,9) [18, 19]. In unserer Studie wurde es erstmals bei NSCLC-Patienten angewandt. Patienten mit NRS und GMS ≥ 3 Punkte hatten ein bis zu 4fach erhöhtes Risiko für schwere postoperative Ereignisse, längere Intensivaufenthalte und verlängerte LOS. Der Cut-off ≥ 3 erwies sich gemäß der Studienlage als unabhängiger Risikofaktor für die Morbidität und die Mortalität [21]. Nicht prädiktiv war der BMI allein, was seine oben genannten Limitationen unterstreicht [1].

Obwohl die Mangelernährungsprävalenz teils mit dem Tumorstadium korrelierte (für Stadium IV a. e. aufgrund der geringen Anzahl nicht aussagekräftig), blieben beide Faktoren unabhängige Prädiktoren für den Langzeitverlauf. Diese Relevanz gilt auch unabhängig von der Durchführung neoadjuvanter Therapien; kongruent zur Studienlage [26]. Die Dominanz des Tumorstadiums kann den Einfluss des Ernährungsstatus überlagern, sodass kein synergistischer Effekt mehr nachweisbar ist.

Für die CAR wurde für unser Kollektiv mangels eines einheitlichen Cut-offs ein ROC-basierter Cut-off von ≥ 0,144 errechnet. Jener war prädiktiv für Unterschiede im RFS und OS; ein um 41 % höheres Mortalitätsrisiko je Anstieg um 1 konnte gezeigt werden. Vergleichend identifizierte eine multizentrische japanische Studie mit älteren Patienten einen Cut-off ≥ 0,106 als prognostisch relevant [15]. International werden vergleichbare Hazard Ratios berichtet [4].

Nach neoadjuvanter Therapie kommt es infolge reduzierter Tumorlast und systemischer Entzündung häufig zu niedrigeren CAR-Werten [14]. Gleichzeitig zeigen sich schlechtere Ergebnisse in NRS und GMS, da therapiebedingte Nebenwirkungen wie Appetit- und Gewichtsverlust das Risiko einer Mangelernährung erhöhen [25].

Während die aktuelle S3-Leitlinie zum Lungenkarzinom zahlreiche klinische Parameter berücksichtigt, fehlen bislang evidenzbasierte Empfehlungen zur präoperativen Erfassung und Behandlung von Mangelernährung [10]. Zwar wurden prähabilitative Konzepte in einigen Studien untersucht, oft fehlen jedoch detaillierte Angaben über Indikation, Art, Dauer und langfristige Endpunkte [23].

Unsere Ergebnisse bieten eine Grundlage zur evidenzbasierten Erfassung des Ernährungsstatus für langfristige Prognosen. Für eine Leitlinienempfehlung bedarf es darüber hinaus einer Diskussion über die klinische Praxis zur ganzheitlicheren Risikoabschätzung von Mangelernährung bei NSCLC-Patienten.

### Limitationen

Aufgrund des retrospektiven, monozentrischen Studiendesigns besteht das Risiko selektiver Dokumentation, wodurch die Generalisierbarkeit der Ergebnisse begrenzt wird. Auch konnten potenzielle Confounder wie Komorbiditäten, präoperative Therapien oder sozioökonomische Faktoren nicht vollständig einbezogen werden. Aufgrund der heterogenen Kohorte waren nur teilweise differenzierte Subgruppenanalysen möglich. Auch die geringe Anzahl neoadjuvanter Therapien maskiert ggf. die tatsächliche Assoziation zum Ernährungsstatus.

Zu berücksichtigen ist zudem, dass bislang weder ein „Goldstandard“ für Ernährungsscreenings etabliert wurde, noch einheitliche CAR-Cut-offs vorliegen. Die CAR bleibt als Surrogatmarker durch immunologische Beeinflussbarkeit für den Ernährungsstatus teils unspezifisch. Zwar erlaubt das ROC-Verfahren eine flexible Anpassung, jedoch ist die Übertragbarkeit auf andere Patientengruppen oder Settings schwierig. Die in vorherigen NSCLC-Studien ROC-basierten Cut-offs zeigen abhängig von Kollektiv und Outcome eine große Range (0,028–0,6) [15, 24]. Unser heterogenes Kollektiv erschwert die externe Validierung zusätzlich; auch weil keine Studien mit vergleichbarem Kollektiv und Ansatz vorliegen.

## Ausblick

Die Untersuchungsergebnisse belegen Mangelernährung als risikobehaftete Erkrankung bei NSCLC-Patienten. Es resultieren wesentliche Auswirkungen auf das postoperative Outcome und den Langzeitverlauf. NRS, GMS und CAR erwiesen sich als komplementäre Instrumente mit Unterschieden in Sensitivität und Prädiktion – eine multiparametrische Risikoeinschätzung ist erforderlich.

Es ergibt sich die klare Implikation, den Ernährungsstatus präoperativ systematisch zu erfassen und Ernährungsscreenings fest in die klinische Routine zu integrieren. In künftigen Studien sollte prospektiv die Verknüpfung von Ernährungsscreenings mit prähabilitativen Ernährungskonzepten in der pneumologischen Onkologie untersucht werden.

## Fazit für die Praxis

- Präoperative Mangelernährung ist bei NSCLC-Patienten häufig (bis zu 65 % [GMS]) und prognostisch relevant.
- Mangelernährung erhöht Komplikationen, LOS, Rezidive und Mortalität bei NSCLC-Patienten.
- Der BMI allein ist nicht aussagekräftig („sarkopenische Adipositas“).
- NRS und GMS identifizieren zuverlässig Risikopatienten; die CAR ergänzt mit hoher prognostischer Aussagekraft (ein Cut-off ≥ 0,144 kann angenommen werden, sollte aber zukünftig weiter evaluiert werden).
- Eine präoperative Risikoabschätzung des Ernährungsstatus mit validierten Instrumenten zur frühzeitigen ernährungstherapeutischen Prähabilitation wird empfohlen.

## Data Availability

Die erhobenen Datensätze können auf begründete Anfrage in anonymisierter Form beim korrespondierenden Autor angefordert werden. Die Daten befinden sich auf einem Datenspeicher in der Klinik für Thoraxchirurgie des Krankenhauses Martha-Maria in Halle/Dölau.
